# Supplementary material for: Micro-scale fusion in dense relativistic nanowire array plasmas
Source: Nat Commun. 2018 Mar 14;9:1077. doi: 10.1038/s41467-018-03445-z (PMC5852030; doi:10.1038/s41467-018-03445-z)
Supplement: Supplementary file 1 — Supplementary Information [file 41467_2018_3445_MOESM1_ESM.pdf]

Micro-Scale Fusion in Dense Relativistic Nanowire Array Plasmas

Curtis et al.

Supplemental information

Supplementary Note 1: Experimental setup

A schematic diagram and photograph of the set up used in the experiment is shown below.

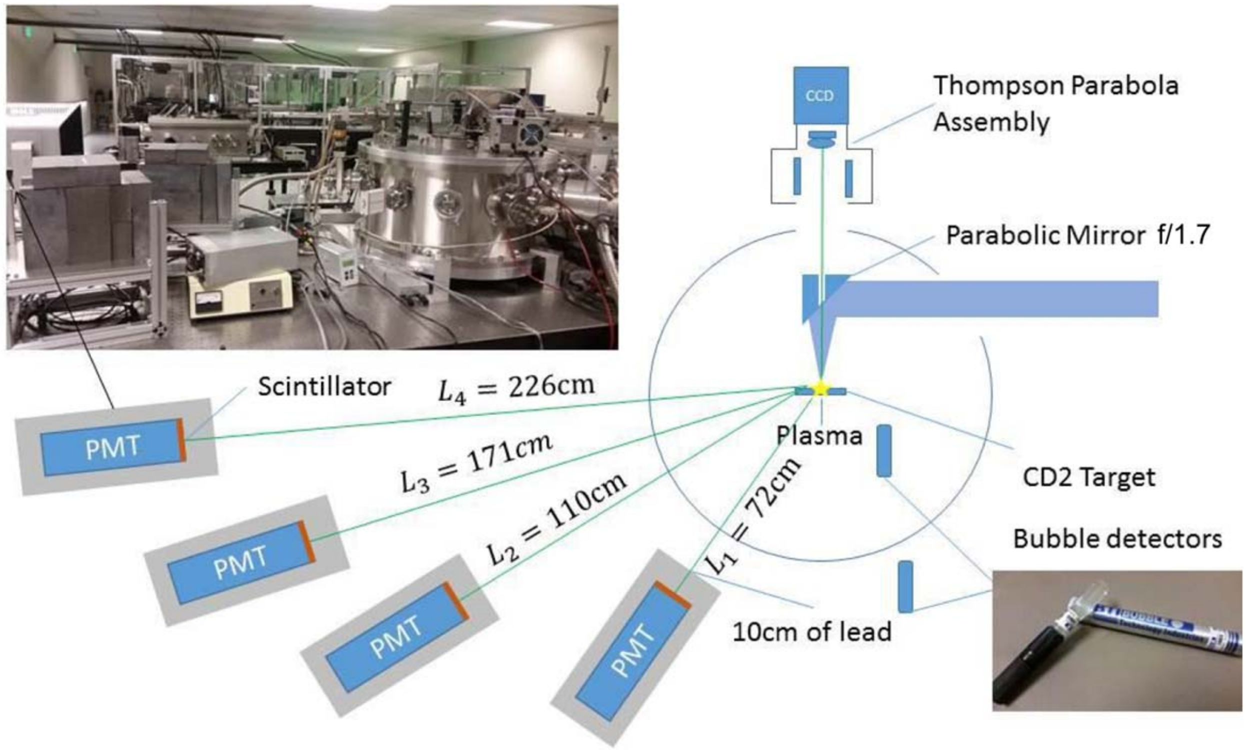

Supplementary Fig 1: **Experimental setup showing target chamber and diagnostics.** The photomultiplier tube (PMT)/scintillator assemblies and the bubble detectors are neutron diagnostics for energy and flux, while the Thomson parabola spectrometer collects ion energy information.

Supplementary Note 2: Thompson parabola supplemental data

A comparison of the Thomson parabola spectra corresponding to array of CH_2 nanowires to an array of CD_2 nanowires. The trace corresponding to a mass to charge ratio of 2 only appears for the deuterated target. The wires are 400 nm diameter.

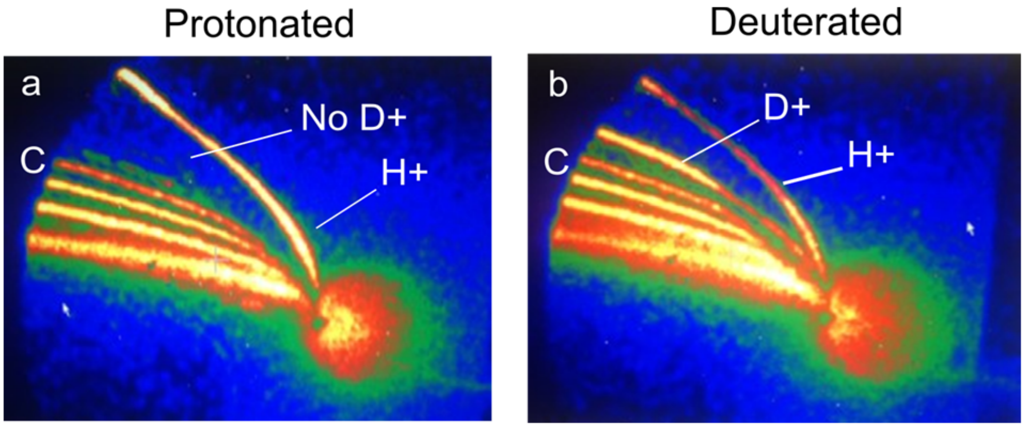

Supplementary Fig 2: **Measured single-shot Thomson parabola energy spectra for protonated and deuterated nanowire arrays**. (a) CH_2 target irradiated at an intensity of 8×10^{19} W cm^{--2}. The traces corresponding to H, and C ions are recorded; (b) array of 400 nm diameter, 5 micron long, CD_2 nanowires. A trace corresponding to D (charge to mass ratio 0.5) is observed.

Supplementary note 3: Measured angular distribution of fast deuterons

The angular distribution of the fast accelerated deuterons measured by positioning CR-39 plates (~ 0.5 cm x 1 cm size) at angles of 22.5 degrees, 45 degrees, 56 degrees, and 67 degrees respect to the target normal. The detectors were placed at different distances to avoid saturation (overlap of holes), and the deuteron flux values were computed correcting for the respective geometric factors. Fractions of the CR-39 plates were covered with 2.5 µm or 4 µm thick Al foils which are transparent to D ions with energy above ~ 270 KeV and ~ 400 KeV respectively, but stop all carbon ions with kinetic energy < 2.5 MeV and < 3.9 MeV respectively. By placing a 2.5 µm Al foil in front of the Thompson parabola we corroborated that all carbon ions are stopped from reaching the CR-39 detector plates. Consequently, only deuterons and H impurity ions are recorded. Fig. S1(a) shows optical microscope photographs of CR-39 plates positioned at three different angles after they were exposed to a single laser shot and developed for two hours. The laser pulse energy on target was 1.38 J.. The angular distribution of the ion flux (number of ions per unit area reaching the detectors) resulting from this

measurement is illustrated in supplementary fig. 4(d). While the focusing parabola did not allow the placement of a CR-39 detector plate in the direction normal to the target, it can be seen that the maximum deuteron flux occurs at the smallest angle respect to the target normal. The ion flux decreases as a function of angle to become nearly extinct for angles > 67 degree. The angular distribution of deuterons measured with both Al foil filters was practically the same.

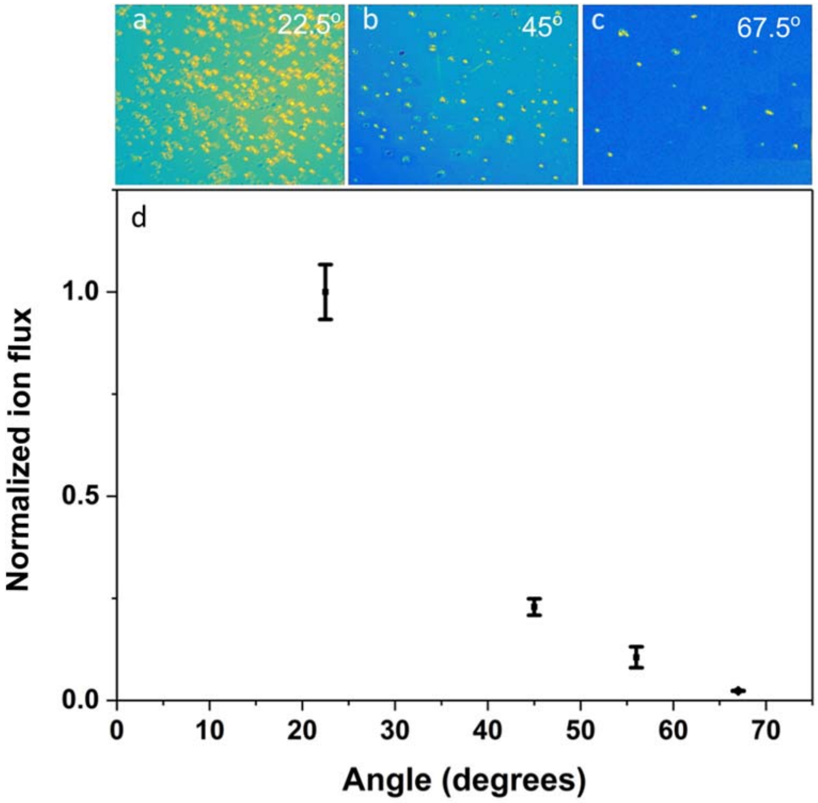

Supplementary Fig 3: **Measured fast deuteron angular distribution.** (a-c) Images of exposed CR-39 plates placed at different angles with respect to the target normal (a-c). The size of each image was adjusted to show the same solid angle. (d) D flux as a function of angle. The nanowires were 200 nm diameter and the laser pulse energy on target was 1.38 J. Error bars represent standard deviation.

Supplementary Note 4: Magnetic field generation and nanowire pinch

The PIC simulations shows that the current produced by the forward acceleration of the electrons in the inter-wire gaps by the laser field via the $\vec{v} \times \vec{B}$ Lorentz force induces a large return through the nanowires. At significantly higher irradiation intensities (eg. 5 x 10^{21} W cm^{-2}) than those used in the experiments reported here, this return current is computed to reach current densities of tens of Mega-amperes per μm^2. This return current will in turn result in the

generation of a strong quasi-static self-generated azimuthal magnetic field which evolution is illustrated in supplementary fig. 4 for the case of CD_2 nanowires irradiated by a laser pulse of 30 fs duration with an intensity of 5 x 10 ^{21} W cm^{-2}. The magnitude of this magnetic field is several GigaGauss. The return current lasts for the duration of the laser pulse. The resulting Lorentz force alters the plasma hydrodynamics pinching the deuterated nanowires into hot plasmas with a peak electron density exceeding 1000 times the critical density (supplementary fig. 4)

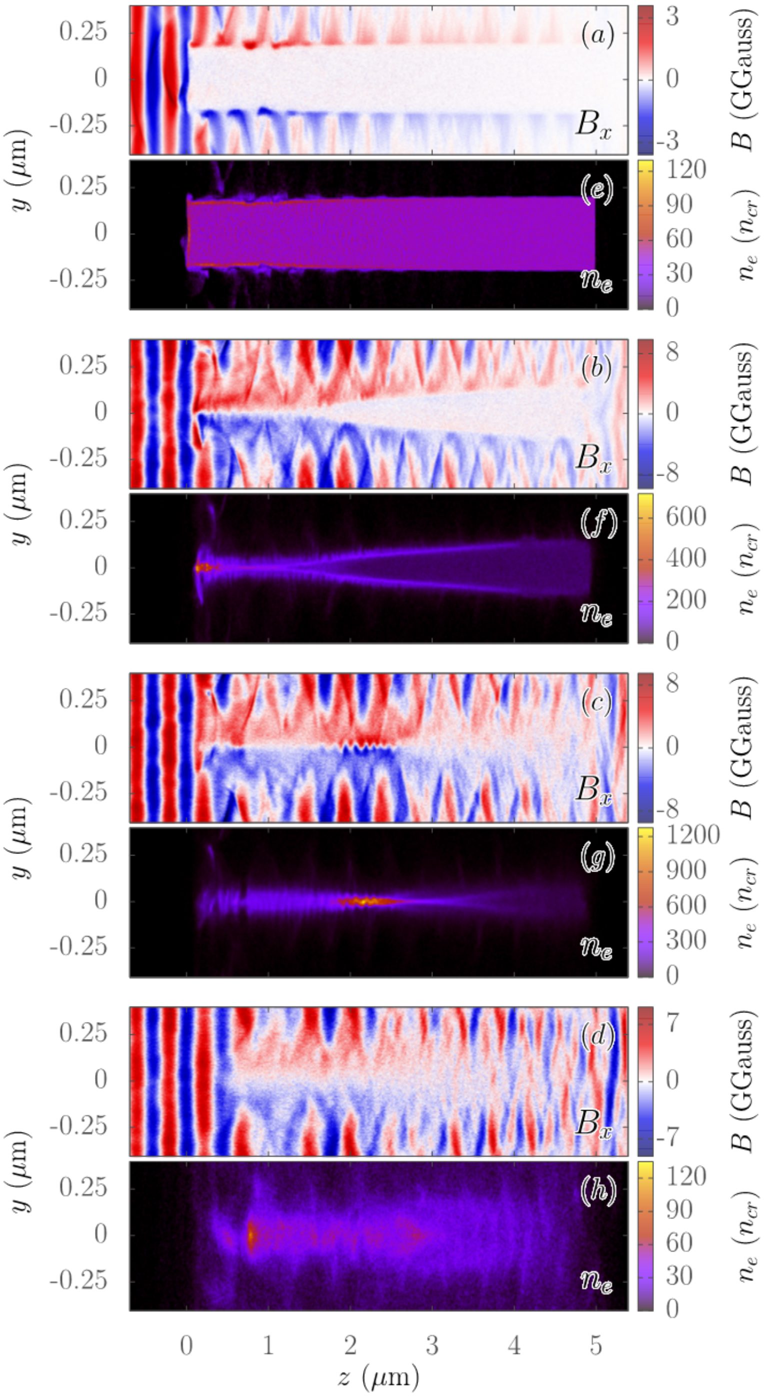

Supplementary Fig 4: PIC simulation showing the spatial distribution of the magnetic field and electron density. Magnetic field (a-d) and electron density (e-h) for four different times (t = -44 fs, t = -10.67 fs, t= 1.33 fs, t = 14.67 fs respectively) with respect to the peak of the 60 fs laser pulse. The 400 nm diameter nanowires are irradiated with an intensity of 5 x 10^{21} W/cm^2 (λ = 400 nm). The return current is observed to generate a strong quasi-static magnetic field that pinches the nanowires**.** The electron density is in units of critical density, n_c = 7 x 10^{21} cm^{-3}.
